# Supplementary material for: Small-Mammal Genomics Highlights Viaducts as Potential Dispersal Conduits for Fragmented Populations
Source: Animals (Basel). 2024 Jan 28;14(3):426. doi: 10.3390/ani14030426 (PMC10854910; doi:10.3390/ani14030426)
Supplement: Supplementary file 1 [file animals-14-00426-s001.zip › animals-2820450-supplementary.pdf]

Supplementary Materials

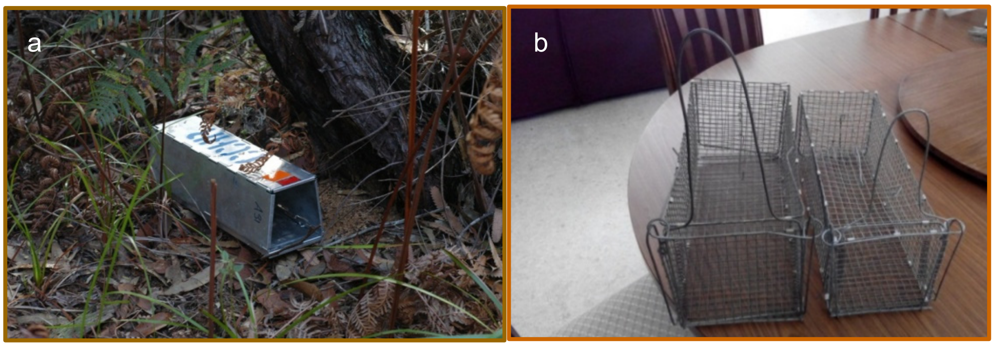

Figure S1. Traps used in this study: (a) Elliott sheet metal trap, (b) Tomahawk wire cage trap (photos by Tabitha Hui).

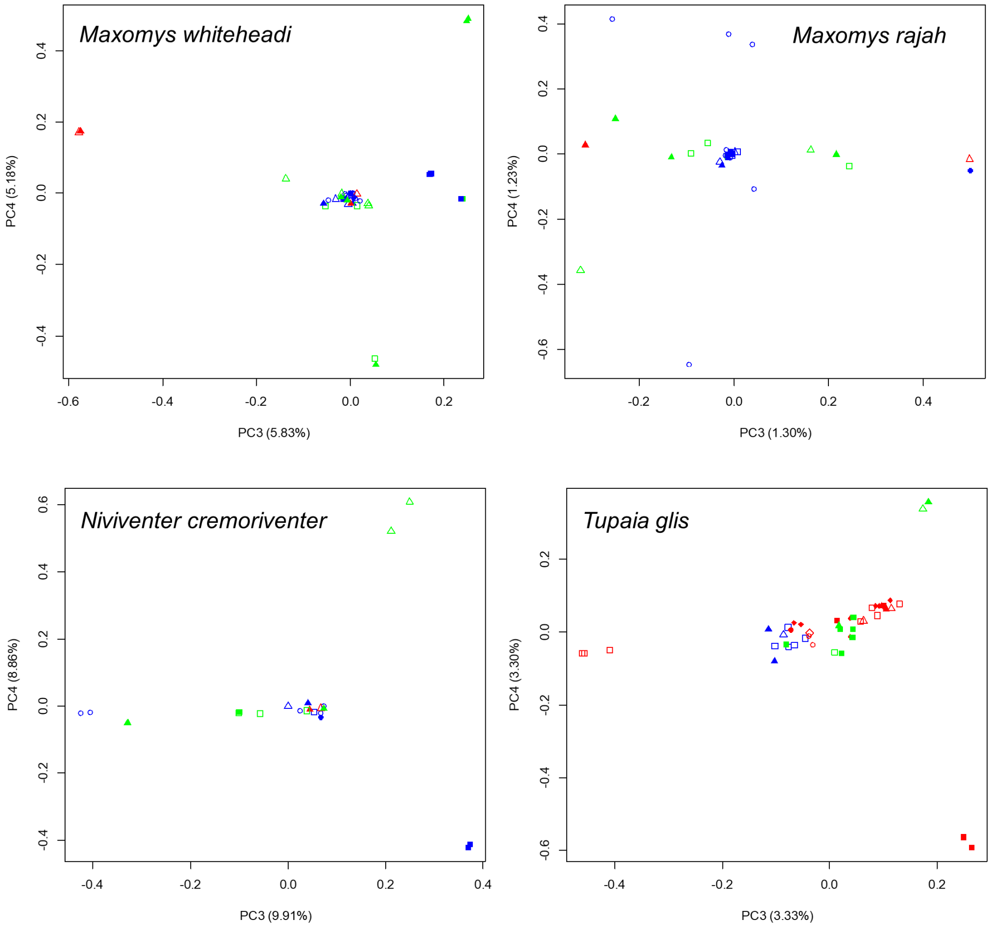

Figure S2. Principal component analysis (axes PC3 and PC4) of genetic differentiation among sampled individuals: (**a**) *Maxomys whiteheadi*, (**b**) *Maxomys rajah*, (**c**) *Niviventer cremoriventer* and (**d**) *Tupaia glis*. Blue: Viaduct sites, Red: Non-viaduct sites, Green: Control sites, Same shape: Same pair, Filled/Open shapes: Opposite sides of the road of the same pair.

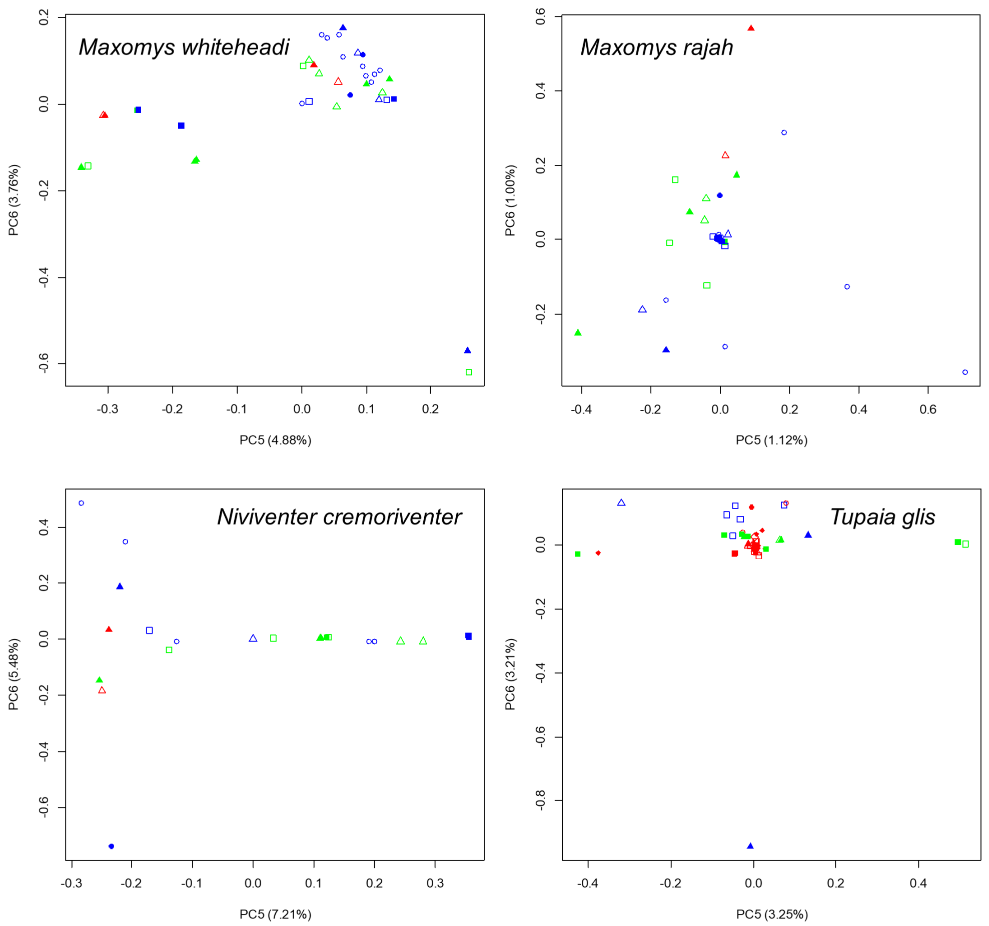

Figure S3. Principal component analysis (axes PC5 and PC6) of genetic differentiation among sampled individuals: (**a**) *Maxomys whiteheadi*, (**b**) *Maxomys rajah*, (**c**) *Niviventer cremoriventer* and (**d**) *Tupaia glis*. Blue: Viaduct sites, Red: Non-viaduct sites, Green: Control sites, Same shape: Same pair, Filled/Open shapes: Opposite sides of the road of the same pair.

Table S1. Individual species samples and sites at which they were captured.

Tupaia glis			*Maxomys rajah*			*Maxomys whiteheadi*			*Niviventer cremoriventer*		
Sample ID	Site	Study area	Sample ID	Site	Study area	Sample ID	Site	Study area	Sample ID	Site	Study area
TG214	NV3N	Kenyir	MR270	NV1S	Kenyir	MW12	NV1S	Kenyir	NC13	V1S	Kenyir
TG216	NV2N	Kenyir	MR274	NV1S	Kenyir	MW20	NV1N	Kenyir	NC14	C2S	Kenyir
TG218	NV2N	Kenyir	MR281	C2S	Kenyir	MW206	C3N	Kenyir	NC205	C3N	Kenyir
TG220	NV3N	Kenyir	MR284	C2S	Kenyir	MW211	V2N	Kenyir	NC239	V3N	Kenyir
TG221	NV3N	Kenyir	MR295	NV1N	Kenyir	MW233	NV2S	Kenyir	NC249	V3N	Kenyir
TG225	C2S	Kenyir	MR79	C2S	Kenyir	MW236	NV3S	Kenyir	NC252	V2N	Kenyir
TG227	NV2S	Kenyir	MRE38	C2N	Kenyir	MW254	V2S	Kenyir	NC268	C3N	Kenyir
TG229	NV3S	Kenyir	MR107	C4E	Sungai Yu	MW276	NV3S	Kenyir	NC272	C3N	Kenyir
TG232	C2N	Kenyir	MR108	C5W	Sungai Yu	MW298	C3N	Kenyir	NC275	C3S	Kenyir
TG250	C1S	Kenyir	MR109	C5E	Sungai Yu	MW46	NV2N	Kenyir	NC278	C2S	Kenyir
TG255	C1S	Kenyir	MR113	C5E	Sungai Yu	MW61	NV3S	Kenyir	NC285	C2N	Kenyir
TG277	NV3N	Kenyir	MR119	C5E	Sungai Yu	MWE27	C3S	Kenyir	NC287	V3N	Kenyir
TG283	C2S	Kenyir	MR127	NV4E	Sungai Yu	MWE28	C3N	Kenyir	NC289	V2S	Kenyir
TG50	NV3S	Kenyir	MR148	NV4E	Sungai Yu	MWE41	NV3S	Kenyir	NC291	V3N	Kenyir
TG51	NV3N	Kenyir	MR151	NV4W	Sungai Yu	MWE43	NV3S	Kenyir	NC293	V3S	Kenyir
TG55	NV3S	Kenyir	MR152	NV4E	Sungai Yu	MWX2022	NV3N	Kenyir	NC4	V1N	Kenyir

TG56	NV3N	Kenyir	MR154	V4W	Sungai Yu	MW114	C5W	Sungai Yu	NC45	V2S	Kenyir
TG59	NV3S	Kenyir	MR185	C5W	Sungai Yu	MW124	V6E	Sungai Yu	NC65	C3S	Kenyir
TG71	C2S	Kenyir	MR191	NV4E	Sungai Yu	MW133	NV5W	Sungai Yu	NC99	V3S	Kenyir
TG72	C2S	Kenyir	MR194	V4E	Sungai Yu	MW134	NV6E	Sungai Yu	NCE44	C2N	Kenyir
TG75	C2S	Kenyir	MR195	NV4W	Sungai Yu	MW136	NV5W	Sungai Yu	NC143	NV5N	Sungai Yu
TG78	C2S	Kenyir	MR197	NV4E	Sungai Yu	MW137	NV5W	Sungai Yu	NC316	NV5E	Sungai Yu
TGE37	NV3N	Kenyir	MR199	NV4W	Sungai Yu	MW140	NV5E	Sungai Yu			
TGE39	NV2S	Kenyir	MR300	NV4W	Sungai Yu	MW144	NV5E	Sungai Yu			
TGX2017	C1N	Kenyir	MR303	V4W	Sungai Yu	MW146	V4W	Sungai Yu			
TG121	NV4W	Sungai Yu	MR305	C4E	Sungai Yu	MW167R	C4E	Sungai Yu			
TG126	NV4E	Sungai Yu	MR306	C4E	Sungai Yu	MW168R	C4E	Sungai Yu			
TG128	NV4E	Sungai Yu	MR322	C5E	Sungai Yu	MW173	NV5E	Sungai Yu			
TG135	NV5W	Sungai Yu	MR323	C5E	Sungai Yu	MW177	V6W	Sungai Yu			
TG138	NV5W	Sungai Yu	MR324	V4E	Sungai Yu	MW186	NV5W	Sungai Yu			
TG139	NV5E	Sungai Yu	MR328	I4W	Sungai Yu	MW189	V4W	Sungai Yu			
TG142	NV5W	Sungai Yu	MR339	I5E	Sungai Yu	MW301	V4E	Sungai Yu			
TG157	V4E	Sungai Yu				MW304	C4W	Sungai Yu			
TG161	V5E	Sungai Yu				MW309	C4W	Sungai Yu			
TG163	V5E	Sungai Yu				MW333	V6E	Sungai Yu			
TG164	V5E	Sungai Yu				MW335	V6W	Sungai Yu			
TG166	V5E	Sungai Yu				MW336	V6E	Sungai Yu			
TG183	NV5W	Sungai Yu				MW340	C5E	Sungai Yu			
TG184	NV5W	Sungai Yu				MW342	C5E	Sungai Yu			
TG188	V4W	Sungai Yu									
TG317	NV5W	Sungai Yu									
TG318	NV5W	Sungai Yu									
TG326	V4W	Sungai Yu									
TG329	V5E	Sungai Yu									
TG338	NV5W	Sungai Yu									

* Last letter of site names refers to site being N-north, S-south, E-east, or W-west of the highway.

Table S2. Number of SNPs at each stage of filtering.

			Number of SNPs							
**Minimum allele frequency**			**No filtering**	**<5%**	**No filtering**	**<5%**	**No filtering**	**<5%**	**No filtering**	**<5%**
**Linkage**			**Linkage**		**No linkage**		**Linkage**		**No linkage**	
**Missingness**			**10% missing data**				**0% missing data**			
	**No. of individuals**	**Raw SNPs**								
Maxomys whiteheadi	Total: 39<br>Kenyir: 16<br>Sungai Yu: 23	824,286	96,264	44,909	66,469	30,638	726	108	272	91
Maxomys rajah	Total: 32<br>Kenyir: 7<br>Sungai Yu: 25	945,781	8,057	4,427	4,297	1,728	1,532	740	849	275
Niviventer cremoriventer	Total: 22<br>Kenyir: 20<br>Sungai Yu: 2	499,093	116,617	68,639	72,220	46,913	1,588	331	659	290
Tupaia glis	Total: 45<br>Kenyir: 25<br>Sungai Yu: 20	1,228,912	349,608	157,390	247,183	103,993	184,286	74,711	137,455	53,557
